# Challenges of Residency Training at the Center of the COVID-19 Pandemic in Wuhan, China

**DOI:** 10.15694/mep.2020.000224.1

**Published:** 2020-10-08

**Authors:** Zhanghong Lu, Stanley Hamstra, Jonathan Lio

**Affiliations:** 1 Renmin Hospital of Wuhan University; 2 Accreditation Council for Graduate Medical Education; 3 University of Chicago

**Keywords:** Postgraduate medical education, COVID-19, international medical education

## Abstract

This article was migrated. The article was marked as recommended.

Soon after the identification of the novel coronavirus in Wuhan, the city experienced a massive outbreak of COVID-19 that severely strained the existing health system. All of the city’s teaching hospitals repurposed buildings for COVID-19 patients, and recruited physicians and trainees of all backgrounds to care of these patients. The authors discuss some of the challenges faced by residency training programs during this period and how they were addressed.

## Introduction

On December 31, 2019, the World Health Organization Country Office received report of a pneumonia of unknown etiology detected in Wuhan, China. Soon after the identification of the new coronavirus and human-to-human transmission, the city locked down to contain its spread on January 23. By the time lockdown was lifted 76 days later, there had been 50,008 infections from Covid-19 and 2,574 deaths in Wuhan ( Health Commission of Hubei Province, 2020). During this time, the number of infections severely strained the health system, as health facilities expanded beyond maximum capacity and the city constructed new hospital buildings in record time. Medical education continued, but residency training programs faced serious challenges under the rapidly escalating outbreak. We describe some of these challenges and how they were addressed.

## Changes to the Nature of Medical Work

In the early period of the outbreak, Wuhan faced a shortage of medical resources, lack of personal protective equipment (PPE), and massive patient volumes. The government designated forty-eight hospitals, including all of the city’s teaching hospitals, as Covid-19-care hospitals and repurposed entire buildings for Covid-19 patients. Physicians and nurses of different specialty backgrounds worked together to treat Covid-19 patients, including 38,478 physicians and nurses who came from other provinces to support Wuhan ( National Health Commission of the People’s Republic of China, 2020). Few hospitals could accept non-Covid-19 patients with other medical issues during the outbreak. Meanwhile, residency program directors and clinical educators, already with little protected time before the outbreak, had even less bandwidth for anything other than clinical care.

## Resident Stress and Well-Being

Confronting a novel infectious disease with a lack of knowledge and expertise, resident physicians experienced a high incidence of anxiety and depressive symptoms ( Chen *et al.*, 2020). Like other licensed physicians, residents were drafted to practice in difficult settings outside their training background, sometimes with decreased supervision. Strict implementation of social distancing policies disrupted residents’ usual social networks and many had difficulty with isolation.

## Risk of Infection During Clinical Practice

In addition to challenges posed by an overloaded medical system and stress from working with anxious patients, resident physicians faced higher infection risks of Covid-19 compared to the public ( Team, 2020). Moreover, if resident physicians were infected, most did not have family members near since they were transplants to Wuhan. A major teaching hospital reported that out of 1061 residents, 105 residents were furloughed for investigation of coronavirus in the workplace, and 11 residents were confirmed with novel coronavirus pneumonia (NCP). The infections mainly occurred in January when human-to-human transmission had not yet been identified and there was shortage of PPE.

## Limitations of Educational Activities

Another consequence of social distancing policies was the prohibition of congregate educational activities. Plans made months or years in advance had to be delayed, adjusted, or cancelled. Due to changes in hospitals’ departmental structures, training programs suspended clinical rotations, and residents were assigned to fixed positions. Certain residents who had left the city before shutdown for Spring Festival vacation were furloughed as they could not return to Wuhan until several months later.

## Ensuring the Safety and Well-Being of Resident Physicians

As there was a high risk of infection for physicians, we needed dedicated personnel to monitor the health and well-being of our resident physicians. At Renmin Hospital of Wuhan University, we designated one administrator to monitor the health and infection status of residents, and another to coordinate resources (e.g. food, daily necessities, medications, financial assistance, counseling) for resident physicians. Residents were required to report if they had close contact with NCP cases without adequate protection, and if they had any Covid-19-related symptoms. All tests and treatment for NCP were free for resident physicians in Wuhan. By April 3, more than 2,500 medical staff in Wuhan, including resident doctors, received a humanitarian assistance grant from the Chinese Red Cross Foundation ( Chinese Red Cross Foundation, 2020).

## Online Learning

Due to social distancing policies, Wuhan residency programs started to use online learning resources to help achieve their curricular goals, which included existing resources on websites, live videos of lectures, and online discussion on video conference platforms. The provincial government also provided online courses for each specialty, and required residents to pass the tests at the end of the courses. Nearly everyone obtained a perfect score on these tests, but a survey of 7,961 resident physicians in the province reported that learner satisfaction was only 60.9% and lecturer satisfaction was 72.1%. Additional training in clinical skills and procedures will be needed after the epidemic when social distancing restrictions are relaxed.

## Adjusting Training Arrangements

Teaching hospitals adjusted the curricular training plan of residents according to the impact of the epidemic. The Chinese Medical Doctors Association added Covid-19 knowledge and skills to the national curriculum for all resident physicians in China, and approved the replacement of rotations required for graduation with experience in Covid-19 wards. Considering the course of the epidemic and policies enacted in response, we divided the Covid-19 epidemic into four periods in Wuhan.

**Early period**: Before January 22 and the identification of human-to-human transmission, residency program administrators followed and disseminated information from government announcements regarding Covid-19.

**Rising period**: From January 22 to February 19, when the number of Covid-19 cases started to increase rapidly and there was a shortage of PPE, clinical rotations for residents and congregate teaching activities were suspended, and the number and frequency of clinical shifts were decreased to reduce the risk of infection and PPE consumption.

**Falling period**: From February 20 to March 5, as the number of active Covid-19 cases began to decline, we started online learning to supplement gaps in the curricular plan.

**Recovery period**: After March 6, Wuhan city began to implement a plan to return to normal operations. For residency programs this included planning for normal clinical rotations, recruitment for incoming residents, and incorporating online learning into resident training and faculty development curricula.

Residents in Wuhan will be able to graduate on time as long as they continue to pass their end-of-rotation exams and their national graduation exams. Two of the four major university-affiliated teaching hospitals in Wuhan have implemented a competency-based education system due to a partnership with University of Chicago, and achievement of target milestones will be monitored for each class of residents. Other teaching hospitals may benefit from enacting a similar system to ensure their graduates are ready for practice ( Hall *et al.*, 2020).

## Conclusion

During the Covid-19 outbreak in Wuhan, teaching hospitals faced an overwhelming number of NCP patients, which altered the nature of resident physicians’ clinical and educational activities, and exposed them to higher risks of infection. Residency training programs attempted to address these challenges by coordinating resources for the health and well-being of residents, procuring online learning content, and adjusting the training curriculum based on the course of the epidemic. We have learned through painful experience the necessity of making contingency training plans for the next possible outbreak. As other nations deal with the increased demands on healthcare resources during the COVID-19 pandemic, as well as maintain adequate standards of training for residents, we hope our experience will help to identify and manage some of the unique challenges to be overcome.

## Take Home Messages

• The outbreak of COVID19 in Wuhan severely strained the health system, disrupting residency training programs and placing residents on the frontlines in managing patients with infection.
• Residency training programs should provide dedicated resources for the health and wellbeing of residents in case of COVID-19 outbreaks.
• Contingency training plans should include online learning and ensure that residents graduate with the competencies necessary for practice.

## Notes On Contributors

**Zhanghong Lu** is Director of Graduate Medical Education, Renmin Hospital of Wuhan University, Wuhan, China. ORCID ID: https://orcid.org/0000-0001-6038-1958

**Stanley Hamstra** is Vice President of Milestone Research and Evaluation, Accreditation Council for Graduate Medical Education, Chicago, IL. ORCID ID: https://orcid.org/0000-0002-0680-366X

**Jonathan Lio** is Assistant Professor, Department of Medicine, University of Chicago, Chicago, IL. ORCID ID: https://orcid.org/0000-0002-3723-2767
