# Supplementary material for: Challenges of Residency Training at the Center of the COVID-19 Pandemic in Wuhan, China
Source: MedEdPublish (2016). 2020 Oct 8;9:224. [Version 1] doi: 10.15694/mep.2020.000224.1 (PMC10699365; doi:10.15694/mep.2020.000224.1)
Supplement: Peer review history

# Reviewer report: Reviewer response for version 1

**DOI:** 10.21956/mep.20167.r31166

**Published:** 2021-02-16

**Authors:** Richard Hays

**Affiliations:** 1 James Cook University

This review has been migrated. The reviewer awarded 4 stars out of 5 This article describes how the health and postgraduate residency training systems responded during the first wave of the 2020 pandemic, right in the heart of the first large outbreak. By the time it was published, many other centres could have (and many have) written a similar story. It is interesting that there are so many similarities in responses to such a major disruptor. I agree that a follow up article on what had to change more permanently would be of interest now, as specialty training has still not recovered fully.

Reviewer Expertise:

NA

No decision status is available

# Reviewer report: Reviewer response for version 1

**DOI:** 10.21956/mep.20167.r31165

**Published:** 2020-12-20

**Authors:** Ken Masters

**Affiliations:** 1 Sultan Qaboos University

This review has been migrated. The reviewer awarded 4 stars out of 5 An interesting article with a unique perspective from Ground Zero of the COVID-19 pandemic. With little warning and no other examples and models of dealing with the pandemic (apart from outbreaks of other viruses), the medical teaching institutions had to implement emergency procedures. Most other medical schools had months to prepare for the pandemic, and still found the process extremely disruptive. Most importantly, though, although they have learned (and can teach) valuable lessons, the one question they cannot answer is: “How would we cope if we were at Ground Zero?” This paper gives some first-hand insight into the scale of the problem.Perhaps my only criticism of the paper is that it should have more detail, especially about “reasonable” assumptions and actions that turned out to be wrong or harmful, and also of the serendipitous or “by-chance” decisions and events that one might not normally consider important that turned out to be important. I say this only because I believe that these are the kinds of things that are forgotten, and yet could provide invaluable insight for the future.Perhaps the authors may wish to expand on these in a Version 2 of the paper. Alternately, if time allows, they may consider writing a whole new paper that gives a far more detailed account. We do not know where and when the next outbreak of a virus will be, but we know for certain that it will happen. The authors’ unique experience could prove crucial to our coping with it.

Reviewer Expertise:

NA

No decision status is available
